# Developmental Differences in Left Ventricular Strain in IUGR vs. Control Children the First Three Months of Life

**DOI:** 10.1007/s00246-022-02850-y

**Published:** 2022-03-25

**Authors:** Olov Änghagen, Jan Engvall, Tomas Gottvall, Nina Nelson, Eva Nylander, Peter Bang

**Affiliations:** 1 Crown Princess Victoria’s Child and Youth Hospital, and Department of Biomedical and Clinical Sciences, Linköping University, Linköping, Sweden; 2 Department of Clinical Physiology in Linköping, and Department of Health, Medicine and Caring Sciences, Linköping University, Linköping, Sweden; 3 Department of Obstetrics and Gynaecology in Linköping, and Department of Biomedical and Clinical Sciences, Linköping University, Linköping, Sweden; 4 Center for Medical Image Science and Visualization (CMIV), Linköping University, Linköping, Sweden; 5 National Highly Specialized Care, Karolinska University Hospital Stockholm, Stockholm, Sweden; 6 Division of Children’s and Women’s Health, Department of Biomedical and Clinical Sciences, Faculty of Health Sciences, Linköping University, Linköping, Sweden

**Keywords:** Cardiac strain, Cardiac function, Fetal growth retardation (FGR), Intra-uterine growth restriction (IUGR), Infant

## Abstract

**Background:**

Intrauterine growth restriction (IUGR) may directly affect cardiovascular function in early life. Longitudinal data on left ventricular longitudinal strain (LVLS), a key measure of cardiac function independent of body size, is not available. We hypothesize impaired cardiac function among IUGR newborns and persistence of the impairment until age 3 months.

**Method:**

This is a prospective cohort study of consecutive pregnancies where IUGR was identified at 18–38 weeks gestational age (GA) with healthy controls randomly selected at 18–20 weeks GA. Echocardiograms were performed at birth and at age 3–4 months, and then compared.

**Results:**

At birth, mean (SD) LVLS did not differ between the IUGR group [*N* = 19; − 15.76 (3.12) %] and controls [*N* = 35; − 15.53 (3.56) %]. The IUGR group demonstrated no significant change in LVLS at age 3–4 months [− 17.80 (3.82) %], while the control group [− 20.91 (3.31) %] showed a significant increase (*P* < 0.001). Thus, LVLS was lower in the IUGR group at age 3–4 months (*P* = 0.003).

**Conclusion:**

The lack of increase in LVLS may suggest that IUGR has a direct impact on cardiac function as early as during the first months of life.

*Trial registration* Clinical trials.gov Identifier: NCT02583763, registration October 22, 2015. Retrospectively registered September 2014–October 2015, thereafter, registered prospectively.

## Impact Statement

- No change in left ventricular longitudinal strain (LVLS) was observed among IUGR infants between birth and age 3–4 months.
- LVLS significantly increased in controls during the same period, resulting in the finding of lower LVLS among IUGR infants compared with controls at age 3–4 months.
- Lack of increase in LVLS among IUGR infants may suggest an impact on cardiac function as early as the first few months of life.

## Introduction

Low birthweight is associated with development of ischemic heart disease, hypertension, and type 2 diabetes later in life [1]. Cardiovascular disease may result from fetal metabolic programming, leading to early development of insulin resistance [2]. Intrauterine growth restriction (IUGR) may also have a direct impact on cardiovascular function in the fetus, neonate, and young child [3–6]. IUGR is common among children born preterm [7] and preterm birth itself increases the risk of clinical heart failure during childhood and preadolescence [8], while also being a risk factor for cardiovascular disease later in life [9].

IUGR neonates have smaller, more spherically shaped hearts at birth than do controls [10, 11]. These changes persist at both one and six months post term [12] and may last up to 8–12 years of age [13], one study describes these anatomical differences at one month post-term, but not at six months or beyond [4].

Echocardiographic parameters reported from studies of the fetus and newborns with IUGR demonstrate a difference in left ventricular stroke volume, cardiac output, and the myocardial performance index between IUGR neonates and controls as early as the first days after birth. Diastolic left ventricular function is affected by IUGR, showing a tendency toward an elevated ratio between early mitral inflow velocity and mitral annular early diastolic velocity, as well as impaired left ventricular isovolumetric relaxation time [14, 15].

Children born preterm show changes in both cardiac morphology and cardiac function similar to those described among IUGR children at birth and in childhood [4, 5].

Most prior studies of left ventricular systolic function in IUGR children have not demonstrated alterations in ejection fraction (EF) [3] while echocardiographic determinations of longitudinal myocardial strain have shown changes associated with IUGR before and after birth [16, 17]. Strain, quantified as deformation of the myocardium expressed as a percentage of uncontracted size, has been proposed as a more sensitive parameter for detection of subclinical left ventricular dysfunction in an array of other conditions [18–20]. IUGR infants demonstrate less LVLS one week post-term than age-matched appropriate for gestational age (AGA) controls [21], suggesting compromised postnatal systolic function in the former group. Lower LVLS was also observed in IUGR-born children at 8–12 years of age compared with AGA controls [13]. Infants born extremely preterm show a gradual increase in LVLS from birth until a few weeks before their estimated due date [22].

In normal pregnancy, fetal LVLS does not change significantly with gestational age (GA) [23]. Systematic meta-analysis of left ventricular strain based on aggregated cross-sectional data has established reference ranges for newborns [24] and for children and adolescents [25]. Children aged one to 18 years show a minimal yet significant decrease in LVLS with age [26].

Thus, previous cross-sectional data indicate that IUGR infants, often born preterm as well, have impaired cardiac function involving a variety of morphological and echocardiographic parameters at birth and during childhood compared with unaffected infants. LVLS varies with age, although changes in LVLS with repeated examinations of the same cohort over time have not been reported.

Among fetuses identified as having IUGR, we studied cardiac function in the newborn and longitudinally in the growing child with assessment of LVLS and other echocardiographic parameters. Here, we present data obtained at birth and at age three months and compare them with data from controls from normal pregnancies. Our overarching hypothesis is that cardiac function is impaired in the IUGR newborn and that this impairment persists through the first 3 months of life.

## Subjects and Methods

### Subjects

This is a prospective cohort study of consecutive IUGR pregnancies, as defined by a fetal weight at least 2.5 SD under or 22% lower than predicted fetal weight for GA [27], and/or as a 10% or greater drop in fetal growth velocity based on the weight that would be predicted from the most recent ultrasound examination. Such pregnancies were identified from routine fetal ultrasound examinations performed during the interval from GA 18–20 weeks and up to 38 weeks, as part of standard clinical care at the tertiary referral university center in Linköping, Sweden. The goal was to include a total of 20 IUGR pregnancies. Inclusion was based on ultrasound-confirmed IUGR as defined above.

From pregnancies where fetal weight was estimated to be within normal limits [at 18 weeks a mean (SD) of 196 (20) g and at 20 weeks 293 (35) g] [27] during routine ultrasound examinations at GA 18–20 weeks, we randomly selected controls at regular intervals during the inclusion period, aiming for a total of 40 controls. Two prenatal examinations, separated by a four-week interval, were performed starting at week 28. The inclusion period ranged from Sept. 2014 to June 2018.

Exclusion criteria included significant malformations, twin pregnancy, intrauterine infections during pregnancy, and serious maternal disease requiring treatment, as well as participation in any interventional study.

### Protocol

Echocardiography was performed 12–72 h and 3–4 months after birth in both IUGR and control infants. Standardized 4-chamber cine loops were recorded for blinded off-line analysis (Vivid E9 and E 95, GE Healthcare Horten, Norway). Velocity vector imaging v. 2.0 (VVI, Siemens Healthcare, Erlangen Germany) was used to determine cardiac motion by tracking the grey scale image.

### Measurements

Images of high-technical quality were selected from each examination and anonymized. Loops of 1–3 beats were analyzed three times (one observer) and the mean values were calculated. Longitudinal velocity, displacement, and LVLS were assessed tangential to the endocardial outline. When analyzing the 4-chamber cine loops, the left ventricle was divided into six segments; longitudinal velocity and displacement were calculated from the basal segments. LVLS was calculated for all six segments, from which the average of the two basal segments and the two middle segments was reported as LVLS. Apical segments were excluded due to software limitations. Sufficient quality of the 2-chamber and 3-chamber views were not obtained in all subjects. To avoid missing data, we therefore, report LVLS obtained from the 4-chamber view. Data from the 2-and 3-chamber images with good quality did not differ systematically from those obtained in the 4-chamber view.

We follow the EACVI/ASE/Industry Task Force recommendation referring to strain as an absolute value, which means that increasingly negative values were reported as increases in LVLS [28].

The sphericity index (SI) was calculated by measuring left ventricular length in diastole and dividing by maximum diameter in diastole, as obtained from the standard 4-chamber view. Left ventricular mass was calculated from measurements in the parasternal long axis projection using the Devereux formula [29].

Birth weight corrected for GA and postnatal weight SD were calculated by extrapolation using reference values obtained from standard Swedish growth charts [30].

### Reproducibility

To assess interobserver variability, 10 randomly selected subjects were identified—3 IUGR infants and 7 controls. Two operators carried out LVLS measurements on the same images at birth and at 3–4 months of age. The population mean for the two assessors was 17.31 with a mean difference of 0.788 and an intraclass correlation coefficient (ICC) of 0.90, which is consistent with high agreement.

### Data Analysis

Sample size and power calculations were performed based on publications showing annular velocity data [31, 32], obtaining a power of 80% and a significance level of 5%, assuming 20% variation in SD.

The data in this report are presented as a mean (SD) or a median (interquartile range, IQR) as appropriate. Normal distribution was checked with the Shapiro–Wilk test. For comparison independent samples *T* test and paired sample *T* test were used to analyze normally distributed data. Mann–Whitney *U* test and the Wilcoxon Signed Rank test was used for skewed data. The results include data on all subjects remaining at any particular timepoint and were analyzed using IBM SPSS Statistics software, RRID:SCR 019096, (IBM, Armonk, New York, United States).

### Ethical Approval

Ethical approval was obtained from the Regional Ethical Review Board in Linköping Sweden (Ref. No. 2012/257-31). Written informed consent was obtained from the parents prior to inclusion of the infant and the study was registered at clinical trials.gov, Identifier: NCT02583763, registration October 22, 2015. Retrospectively registered September 2014–October 2015, thereafter, registered prospectively.

## Results

**Fig. 1:**
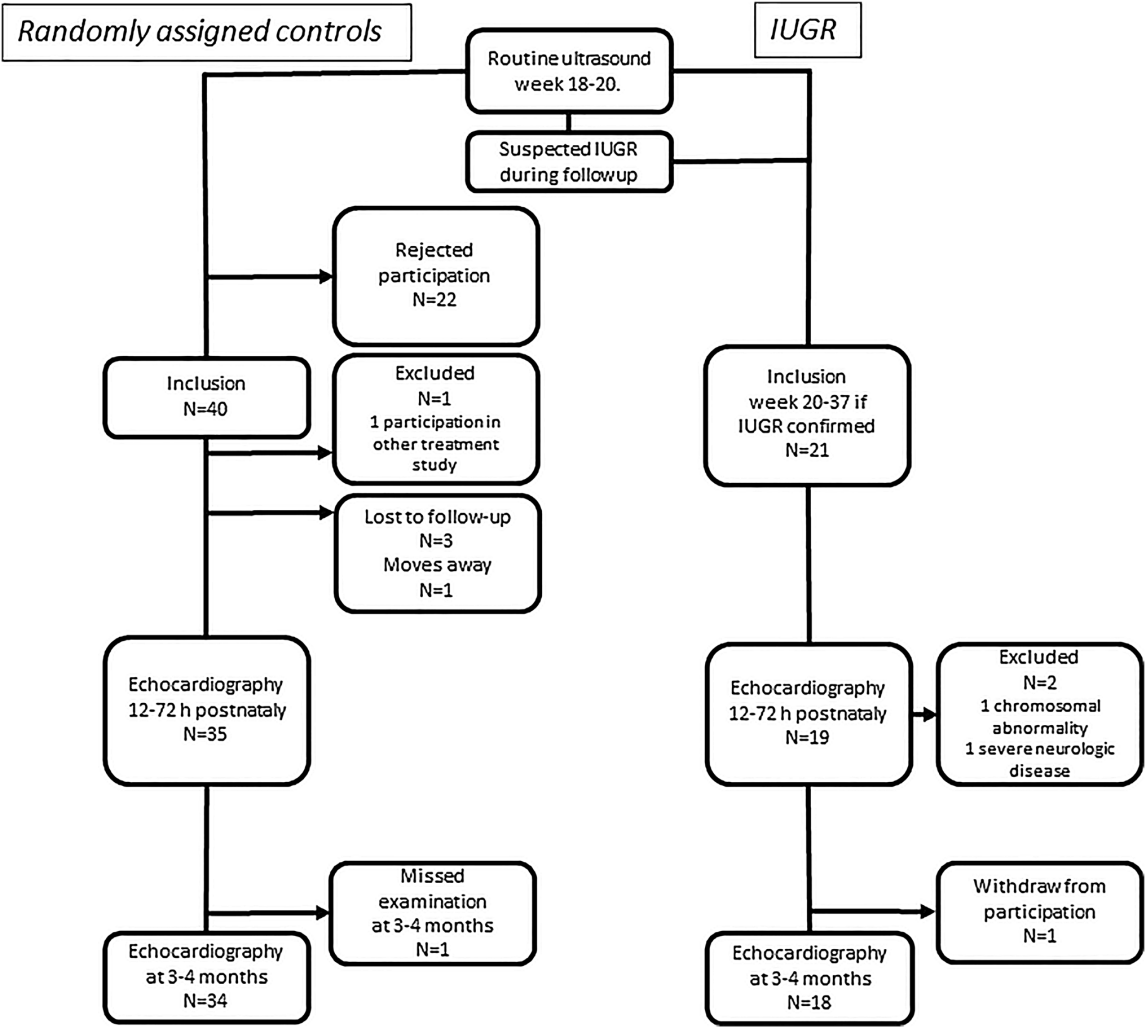
Flow diagram of progress throughout the study

The study included 21 IUGR infants, mean GA 33.3 (range: 29.5–35.2) weeks, of whom 19 were available for follow up (Fig. 1). Of the 40 controls included at a mean GA of 19.3 (18.9–19.7) weeks, 35 were available for follow-up (Fig. 1). Table 1 shows that the IUGR infants demonstrated a mean deviation in weight of -28% (range: − 38 to − 25) at inclusion and remained growth retarded with a mean weight − 2.9 (1.27) SD lower at birth compared with controls, a significant difference. In addition, the IUGR infant group was born at an earlier GA; 6 required ICU admission, although they did not need ventilator treatment or inotropic medication. All but two of the IUGR infants demonstrated catch-up growth with a mean weight gain of 2.2 (1.5) SD, but still had a significantly lower SD in weight when echocardiography was repeated at age 3–4 months. The SD in weight of controls was close to the reference mean on both assessment occasions (Table 1).

**Table 1:** Characteristics of the study group

	IUGR	Control
*Fetus*		
N	19	35
Prenatal weight deviation, %	− 28(− 38 to 25)% †††	− 1.3 (8.9)
Gestational age at 1st ultrasound, weeks	31.8 (5.0)	29.7 (3.2)
*Newborn*		
*N*	19	35
Girls, *N* (%)	9 (47)	17 (49)
Gestational age at birth, weeks	36.4 (3.9)†††	39.7 (1.5)
Birth weight, gram	2077 (773)†††	3415 (381)
Birth weight, SD	− 2.91 (1.27)†††	0.75 (0.13)
Apgar score at 5 min	9.5 (1.43)	9.9 (0.36)
Ventilator, *N* (%)	0 (0)	0 (0)
Intensive care unit, *N* (range of days in ICU)	6 (8–54)	0 (0)
*3–4 months*		
*N*	18	35
Girls, *N* (%)	8 (44)	17 (49)
Weight SDS	− 0.69 (1.46)††	0.3 (0.79)
Weight SDS change from birth to 3–4 months of age	2.22 (1.49)†††	0.4 (0.96)
*Mothers*		
Age at birth, years	31.8 (5.50)	30.0 (5.35)
BMI at birth	27.7 (5.35)	29.5 (2.33)
Prior pregnancies, grava	2.8 (1.78)	2.6 (1.54)
Prior deliveries, para	0.9 (1.41)	1.0 (0.97)
Weight gain during pregnancy, kg	14.5 (13.4)	13.9 (10.6)
Hemoglobin in early pregnancy, g/L	126 (9.63)	125 (9.20)
Proteinuria during pregnancy, *N* (%)	3 (16)	0 (0)
SBP in early pregnancy, mmHg	110 (105–120)	110 (9.0)
DBP in early pregnancy, mmHg	65 (70–75)††	70 (11.7)
SBP prior to delivery, mmHg	120 (110–135)	116 (11.0)
DBP prior to delivery, mmHg	80 (70–85)††	70 (7.5)
Smoking prior to pregnancy, *N* (%)	2 (10)	3 (9)
Smoking during pregnancy, *N* (%)	1 (5)	1 (3)
Alkohol at most once a week, *N* (%)	5 (26)	11 (31)
Alkohol during pregnancy	0	0
Druguse prior pregnancy	0	0
Druguse during pregnancy	0	0

IUGR versus Control

*SBP* systolic blood pressure, *DBP* diastolic blood pressure

*P* value: †  < 0.05, ††  < 0.01, †††  < 0.001

The only difference between IUGR mothers and control mothers was a higher diastolic blood pressure in early pregnancy and prior to delivery in the former group (Table 1).

Initial echocardiographic examinations were performed on IUGR infants 44 (18) hours post-delivery and on controls at 47 (24) hours (Table 2). The 3–4 month examinations of IUGR infants were conducted on average at day 116 (95–137) chronological age (CA) and day 100 (57) gestationally corrected age (GCA). The corresponding examinations on controls were performed on day 116 (23) CA and day 116 (25) GCA.

**Table 2:** Temporal development of echocardiographic measurements

*		Newborn				3–4 months				Newborn vs. 3–4 months	
		IUGR	IUGR adj for left ventricular length	Control	Control adj for left ventricular length	IUGR	IUGR adj for left ventricular length	Control	Control adj for left ventricular length	IUGR	Control
	Chronological age at exam (days)	1.85 (0.75)		1.95 (0.99)		118 (92–134)		116.4 (22.9)			
	Chronological age at exam (hours)	44 (18)		47 (24)		NA		NA			
	Corrected age at exam (days)	NA		NA		99.7 (56.7)		115.6 (25.1)			
	Length (cm)	43 (5.1) †††		50 (1.7)		55 (4.1) †††		61 (2.4)		***	***
	Weight (g)	2077 (773) †††		3415 (381)		4655 (929) †††		6117 (660)		***	***
	BSA (Mosteller)(m^2^)	0.16 (0.039) †††		0.22 (0.015)		0.29 (0.040) †††		0.34 (0.031)		***	***
	Heart rate (Beats per min)	126 (28.1)		122 (19.5)		147 (18.9)		139 (24.0)		*	**
LV	LVEDd (cm)	1.46 (0.20) †††		1.72 (0.14)		1.98 (0.24)		2.14 (0.20)		***	***
	IVSd (cm)	0.25 (0.051) †††		0.33 (0.072)		0.35 (0.051)		0.37 (0.091)		***	
	LVPWd (cm)	0.25 (0.061) †††		0.31 (0.054)		0.36 (0.050)		0.37 (0.080)		***	**
	LV mass (g)	4.42 (1.55)†††		7.38 (1.47)		10.06 (3.45)		11.52 (4.73)		***	***
	LV mass index	28.2 (6.23) ††		33.7 (5.76)		37.67 (9.64) †		35.45 (10.68)		***	
	RWT	0.35 (0.27–0.40)		0.35 (0.32–0.39)		0.36 (0.35–0.38)		0.35 (0.078)		*	
	DL septum (mm)	2.76 (0.79)	0.112 (0.026)	3.16 (0.77)	0.110 (0.028)	4.72 (0.84)††	0.141 (0.030)	5.69 (1.10)	0.154 (0.031)	***/§§	***/§§§
	VL septum systole (cm/s)	2.32 (0.52)†	0.095 (0.018)	2.67 (0.66)	0.087 (0.079–0.11)	3.56 (0.65)†	0.107 (0.024)	4.03 (0.75)	0.109 (0.020)	***	***/§§
	VL septum diastole (cm/s)	− 2.73 (0.66)†	− 0.112 (0.025)	− 3.25(0.8)	− 0.112 (0.029)	− 5.00 (1.07)††	− 0.151 (0.040)	− 6.20 (1.50)	− 0.167 (0.041)	***/§§	***/§§§
	DL lateral wall (mm)	2.48 (1.01)	0.101 (0.039)	2.67 (2.16–3.08)	0.088 (0.075–0.11)	4.1 (1.26)†	0.122 (0.036)	5.17 (1.70)	0.138 (0.044)	***	***/§§§
	VL lateral wall systole (cm/s))	2.28 (0.88)	0.085 (0.06–0.11)	2.45 (0.69)	0.085 (0.025)	2.98 (0.76)††	0.089 (0.021)‡	3.90 (1.12)	0.105 (0.030)	*	***/§§
	VL lateral wall diastole (cm/s)	− 2.72 (1.02)	− 0.111 (0.039)	− 3.14 (1.20)	− 0.110 (0.044)	− 4.71 (1.53)†	− 0.141 (0.047)	− 5.78 (1.75)	− 0.155 (0.047)	***	***/§§§
	Avrage four segment SL (%)	− 15.76 (3.12)		− 15.53 (3.56)		− 17.80 (3.82)††		− 20.92 (3.31)			***
	Avrage SL septal wall (%)	− 15.49 (3.66)		− 16.44 (3.61)		− 18.06 (3.13)††		− 20.29 (3.34)			***
	Avrage SL lateral wall (%)	− 16.02 (4.44)		− 14.61 (5.22)		− 17.55 (6.07)††		− 21.55 (5.03)			***
	Sphericity Index	1.85 (0.17)†		1.74 (0.16)		1.63 (0.16)		1.67 (0.17)		**	–
RV	DL septum (mm)	2.74 (0.94)††	0.155 (0.14–0.20)	3.6 (0.93)	0.125 (0.035)	6.09 (0.90)†	0.182 (0.032)	6.71 (0.89)	0.181 (0.024)	***/§§§	***/§§§
	VL septum systole (cm/s)	2.40 (2.0–2.8)†	0.156 (0.11–0.17)	2.82 (0.62)	0.098 (0.022)	4.49 (0.83)	0.134 (0.026)	4.82 (0.69)	0.130 (0.020)	***/§§§	***/§§§
	VL septum diastole (cm/s)	− 2.88 (1.03)	− 0.117 (− 0.14 to 0.09)	− 3.35 (− 4.32 to 2.60)	− 0.125 (0.050)	− 6.05 (1.25)††	− 0.171 (− 0.20 to 0.15)	− 7.25 (1.44)	− 0.195 (0.039)	***/§§§	***/§§§
	DL free wall (mm)	3.97 (1.76)††	0.161 (0.061)	5.5 (1.73)	0.192 (0.064)	6.56 (2.35)	0.198 (0.076)	6.65 (2.5)	0.175 (0.074)	**	-
	VL free wall systole (cm/s)	3.39 (1.31)†	0.140 (0.051)	4.35 (3.20–5.12)	0.151 (0.052)	5.75 (1.69)	0.172 (0.055)	6.05 (1.98)	0.159 (0.061)	***/§§	**
	VL free wall diastole (cm/s)	− 4.11 (1.95)†	− 0.168 (0.073)	− 5.23 (1.73)	− 0.176 (− 0.24 to 0.12)	− 7.23 (2.23)	− 0.218 (0.075)	− 8.05 (2.98)	− 0.211 (0.089)	***/§	***

Devereaux Formula for LV Mass (g) = 0.8{1.04[([LVEDD + IVSd + PWd]^3^ − LVEDD^3^)]} + 0.6

Relative wall thickness (RWT) = 2 × PWd/LVEDD

Left ventricular mass index = LV mass/BSA

LV sphericity index = length/diameter

BSA body surface area (Mosteller), *LVEDd* left ventricular end diastolic diameter), *IVSd* interventricular septum thickness at end diastole, *LVPWd* left ventricular posterior wall thickness at end diastole, *LV mass* left ventricular mass, *LV* left ventricle, *RV* right ventricle, *DL* displacement longitudinal, *VL* velocity longitudinal, *SL* strain longitudinal

†IUGR versus control, † < 0.05, †† < 0.01, †††  < 0.001

‡IUGR versus Control adj for ventricular length, ‡ < 0.05, ‡‡ < 0.01, ‡‡‡ < 0.001

*Birth versus 3–4 months, * < 0.05, ** < 0.01, *** < 0.001

§Birth versus 3–4 months adj for ventricular length, § < 0.05, §§ < 0.01, §§§ < 0.001

### Left Ventricular Longitudinal Strain

**Fig. 2:**
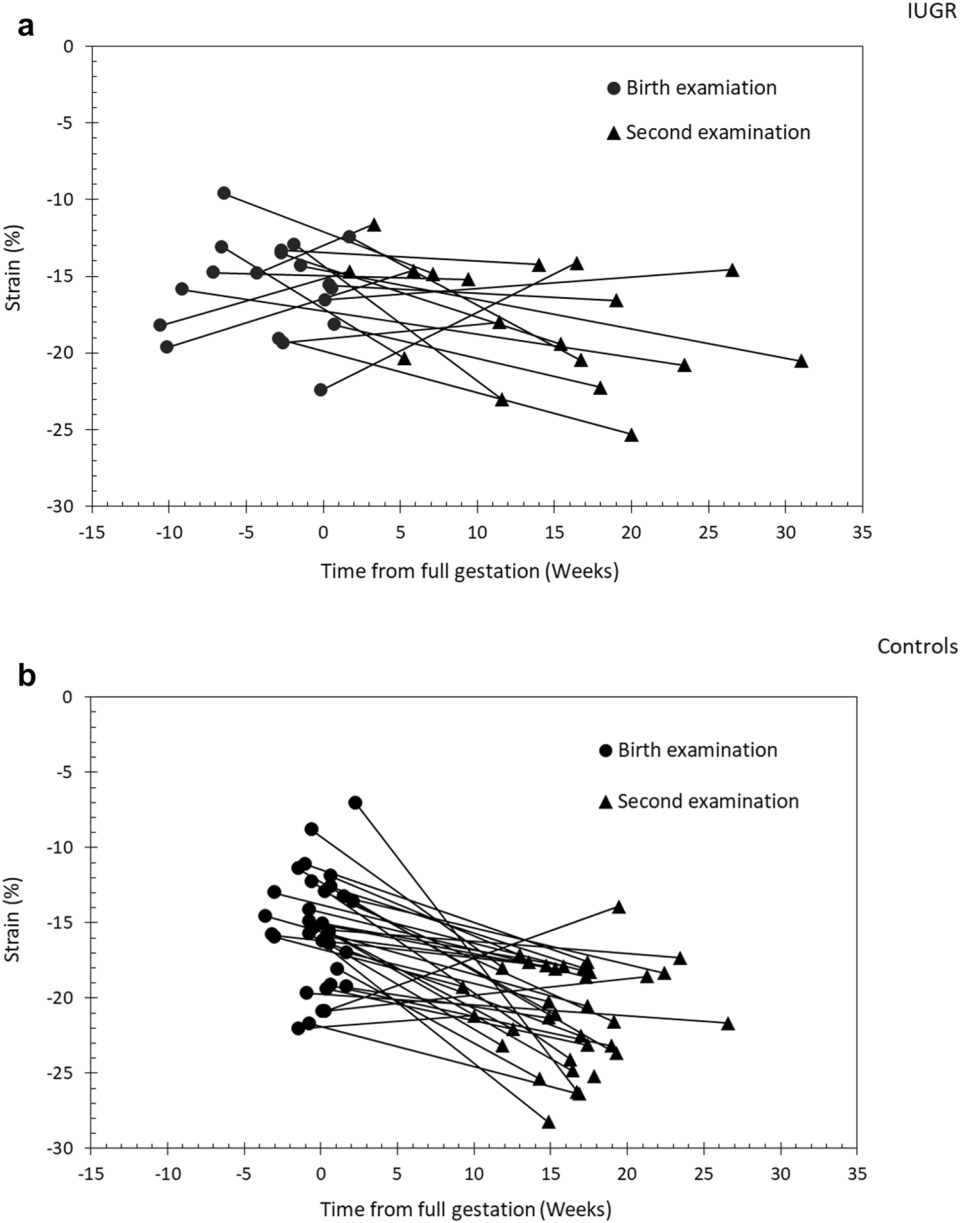
**a** Change in left ventricular longitudinal strain (LVLS) in relation to gestationally corrected age (GCA), IUGR. **b** Change in left ventricular longitudinal strain (LVLS) in relation to gestationally corrected age (GCA), Controls

LVLS did not differ between the groups at birth (Table 2). In IUGR infants, no change in LVLS was observed between birth and age 3–4 months. In contrast, LVLS among controls increased significantly during this period (*P* < 0.001). Thus, LVLS was lower in IUGR infants than in controls (*P* = 0.003) at age 3–4 months. Figure 2a and b show individual plots of LVLS over time in relation to GCA.

To analyze the lack of a normal increase in LVLS in the IUGR group, we performed univariate correlation analysis in all subjects, considering the two groups as a continuum. This showed a positive correlation between the increase in LVLS (birth to 3–4 months) and both prenatal weight deviation at inclusion (*R* = 0.310, *P* = 0.032) and birth weight (*R* = 0.277, *P* = 0.046). In addition, the increase in LVLS (birth to 3–4 months) correlated positively with GA (*R* = 0.296, *P* = 0.033), but not with birth weight standard deviation score (SDS) (*R* = 0.232, *P* = 0.098). We also found that LVLS at age 3–4 months correlated positively with both weight deviation at inclusion (*R* = 0.389, *P* = 0.006) and birthweight (*R* = 0.342, *P* = 0.012), but correlated negatively with weight SDS gain between birth and age 3–4 months (*R* = 0.320, *P* = 0.019). However, LVLS at age 3–4 months also showed a positive correlation with GA at birth (*R* = 0.366, *P* = 0.007). Children in whom LVLS was lower did not differ on an individual basis from the remaining children with increasing LVLS regarding GA, birth weight, birth weight SDS, or weight gain SDS. None of these variables showed significant correlation in a multivariate regression model.

### Heart Dimensions

The left ventricular mass index (LVMI) in the newborn was significantly lower in the IUGR group with smaller hearts (28.2 vs. 33.7 g/m^2^; *P* < 0.01) (Table 2), but after catch-up growth at age 3–4 months the IUGR infants had a larger LVMI (37.7 vs. 35.5 g/m^2^; *P* < 0.05).

The left ventricular sphericity index was higher at birth in IUGR newborns compared with controls, indicating a less spherical ventricle in the IUGR group (*P* < 0.05). However, the sphericity index decreased in the IUGR group and by age 3–4 months there was no longer any difference compared with controls.

### Longitudinal Displacement and Velocity

Left ventricular longitudinal displacement and velocity at birth, corrected for ventricular length, did not differ between the groups with the exception of systolic longitudinal velocity in the lateral wall at age 3–4 months (0.089 vs. 0.105; *P* < 0,01) (Table 2). No change in corrected left ventricular lateral wall displacement and velocity was observed between birth and age 3–4 months in the IUGR group, but such changes were significant among controls. Concerning the right ventricle, no differences were observed between groups either at birth or at age 3–4 months, and no changes occurred over time with the exception of minor differences in the right free wall.

## Discussion

We have observed that LVLS increases in normal non-affected newborns during the first 3–4 months after birth, but such changes were not seen among IUGR infants. This paper is the first to present longitudinal data on normal developmental changes in LVLS during the first 3–4 months of life among infants from non-IUGR pregnancies. Furthermore, our finding that left ventricular systolic deformation in IUGR infants does not increase may indicate a propensity for cardiovascular disease later in adult life. Since the metabolic programming associated with IUGR may require time before heart function is impacted, our findings may suggest that IUGR also has a direct impact on cardiac function as early as during the first months of life.

However, it’s too early to make any clear conclusion about the adult consequences of these early findings and future studies needs to confirm the lack of increase in LVLS in the early life of newborn IUGR children. Longitudinal follow-up of cardiac function into later childhood and adult life is necessary.

Few studies address LVLS among IUGR infants. In the cross-sectional study by Akazawa et al. [21], small for gestational age (SGA) infants demonstrated an approximately 10% lower LVLS than controls at one week of age. In their study, both the SGA and control infants were born slightly preterm (GA around 35 weeks). The SGA infants with IUGR had not been identified prior to birth, but the more impacted group showed a birth weight SD of − 2.6, which was quantitatively similar to the growth retardation seen in our IUGR group. Left ventricular strain was determined by a 16-segment model and examinations were performed approximately 5 days later than in our study, although the exact time was not reported. Our study showed that the IUGR group had a non-significant ≈ 15% increase in LVLS at age 3–4 months, while our controls demonstrated a significant ≈35% increase. However, our study design does not allow us to specify the exact time when this increase occurred.

In preadolescent IUGR children, an 18-segment model demonstrated a significant ≈ 5% lower LVLS compared with controls, suggesting that these changes persist, at least partially, over time [13].

Although this suggests that IUGR affects cardiac function, children with growth restriction are also more prone to preterm birth [33], for which reason it may be difficult to separate the effects of growth restriction from those of preterm birth. The mean GA at birth in our study is 36.4 weeks in IUGR infants and 39.7 weeks in controls. Since the IUGR infants in our study were born more preterm than the controls, the lack of changes in LVLS over time may, to some extent, be related to preterm birth. Although our individual analysis of GA vs changes in left ventricular strain showed that the two most preterm children demonstrated a decrease in LVLS between birth and age 3–4 months, our results also found that IUGR infants born near term showed decreasing LVLS, while IUGR infants born before 37 weeks showed increasing LVLS. Moreover, we were unable to ascertain any other variables, including birth weight, birth weight SDS, or weight gain SDS between birth and age 3–4 months that could identify individuals (IUGR or controls) with decreasing LVLS.

James et al. reported that extremely preterm children (born week 26–30), who were less affected by IUGR than our cohort (12% of the study group below the 10th percentile birth weight SD), had a small but significant increase in LVLS between birth and reaching a GCA of 36 weeks [22]. However, this study is only relevant as a surrogate control for the most preterm IUGR infants in our cohort, given that the mean GA at birth among our IUGR infants was 36.4 weeks. A cross-sectional study determined LVLS among fetuses in utero, showing only a minor increase during the last trimester [23]. However, this information becomes less relevant in the current context due to important changes in hemodynamics and left ventricular function that occur at birth.

The study by Akazawa et al. [21] collected GA-matched controls. Our study design did not allow for collection of GA-matched controls due to the inclusion of IUGR fetuses and non-affected controls and subsequent longitudinal examinations at various timepoints in utero, at birth, and during childhood. Our findings—that both LVLS at age 3–4 months and the increase in LVLS between birth and age 3–4 months show a positive correlation with GA in univariate analysis – support a significant impact of GA on LVLS. Earlier studies have found that GA affects postnatal development of LVLS in the left free wall, where extremely preterm infants (GA 26–30) showed similar LVLS at term-equivalent age compared with controls, but lower LVLS at age 6 months [34]. This observation is also consistent with our finding that LVLS does not correlate with birth weight SDS since this parameter increased with GA, suggesting that low GA is more important than birth weight SDS as a determinant of impaired increase in LVLS during the first three months of life among IUGR infants. On the other hand, Cohen et al. found that both IUGR and prematurity are independently related to subclinical changes in diastolic function with a higher ratio of early mitral inflow velocity to mitral annular early diastolic velocity among preterm infants, both with and without IUGR, but not among term infants without IUGR. The Cohen study found no differences in systolic function among the three groups [4]. Although it may be difficult to fully ascertain the relative importance of IUGR and preterm birth, the two are often associated and cause direct effects on cardiac function. Metabolic programming may also contribute to cardiovascular disease later in adult life and both the metabolic and direct cardiac mechanisms may explain the reduction in LVLS found among adults born both term and preterm when compared with controls, all of which suggest that the early findings persist into adulthood [35].

We noted rapid weight gain during the first few months of life in our IUGR group; catch-up growth has previously been suggested to be an independent risk factor for cardiovascular disease in adult life [36]. We found that children with a more pronounced weight SDS gain demonstrated a less increase in LVLS at 3–4 months, which is in line with the hypothesis that rapid weight gain has a negative impact on cardiac function. It also points to the IUGR children with a more pronounced weight SDS gain being responsible for the lack of LVLS increase in the IUGR group. An association has been demonstrated between adult coronary heart disease and catch-up growth to achieve average or above average weight between ages 7 and 15 years [37]. Postnatal growth up to 6 months of age is more predictive of the risk of developing elevated blood pressure by age 3 years than is birth weight [38]. IUGR infants are at increased risk of developing metabolic syndrome, type 2 diabetes, insulin resistance, and cardiovascular diseases if they experience rapid catch-up growth early in life [39]. Finally, rapid weight gain is commonly seen in children born with IUGR [40–42] and this effect may be related to both IUGR and preterm birth.

Our study also examined heart measurements and found that the controls were born with more spherical ventricles than the IUGR infants and that this difference disappeared at age 3–4 months. This finding contrasts with one previous study that found a more spherical heart shape among IUGR infants at age 1 month [3], while yet another study described preterm infants born with IUGR as having more spherical heart chambers than preterm infants without IUGR at age 1 month [4].

We found that the IUGR-associated differences in myocardial longitudinal velocity and displacement of basal segments were related to cardiac size, as has been previously reported. After correcting for ventricular length, no significant group differences were found other than the lack of change in displacement or velocity in the left lateral ventricular wall among IUGR infants between birth and age 3–4 months; such changes during this time interval were found to be significant in the control group. When comparing average LVLS in the left lateral ventricular wall, a larger difference was noted at age 3–4 months between the IUGR group and controls than any changes seen in the septal wall. Earlier studies have indicated the presence of a more spherical heart and left ventricular dilatation secondary to early cardiac remodeling among IUGR infants [3], one hypothesis states that cardiac remodeling may affect movement of the lateral wall more than the septal wall of the left ventricle.

The strength of our study lies in its design of longitudinal pre- and postnatal examinations of infants who were identified in utero as having or not having IUGR. IUGR pregnancies were diagnosed as early as GA week 20 and up to week 37, thereby representing a spectrum of early to late onset of growth restriction. Very few infants were excluded or lost to follow-up at the 3–4-month visit. However, the number of infants in the IUGR and control groups is limited, which is typical for this kind of study, but this imposes limitations on the ability to analyze subgroups (for example, early vs. late growth restriction).

In summary, the IUGR infants in our study, born slightly preterm on average, demonstrate an attenuated increase in left ventricular longitudinal strain during their first “preterm” months of life.

## Data Availability

Available on reasonable request, to preserve subjects anonymity and integrity.

## Code Availability

Velocity vector imaging v. 2.0 (VVI, Siemens Healthcare, Erlangen Germany). SPSS Statistics software, RRID:SCR_019096, (IBM, Armonk, New York, United States).

## Material Availability

Available on reasonable request, to preserve subjects anonymity and integrity.
